# Predicting Regional Cerebral Blood Flow Using Voxel-Wise Resting-State Functional MRI

**DOI:** 10.3390/brainsci15090908

**Published:** 2025-08-23

**Authors:** Hongjie Ke, Bhim M. Adhikari, Yezhi Pan, David B. Keator, Daniel Amen, Si Gao, Yizhou Ma, Paul M. Thompson, Neda Jahanshad, Jessica A. Turner, Theo G. M. van Erp, Mohammed R. Milad, Jair C. Soares, Vince D. Calhoun, Juergen Dukart, L. Elliot Hong, Tianzhou Ma, Peter Kochunov

**Affiliations:** 1 Department of Epidemiology and Biostatistics, University of Maryland, College Park, MD 20742, USA; kehj@terpmail.umd.edu (H.K.); tma0929@umd.edu (T.M.); 2 Department of Psychiatry and Behavioral Sciences, UTHealth Houston School of Behavioral Health Sciences, University of Texas Health Science Center at Houston, Houston, TX 77054, USA; si.gao@uth.tmc.edu (S.G.); yizhou.ma@uth.tmc.edu (Y.M.); mohammed.r.milad@uth.tmc.edu (M.R.M.); jair.c.soares@uth.tmc.edu (J.C.S.); l.elliot.hong@uth.tmc.edu (L.E.H.); peter.kochunov@uth.tmc.edu (P.K.); 3 Maryland Psychiatric Research Center, Department of Psychiatry, University of Maryland, Baltimore, MD 21228, USA; ypan@som.umaryland.edu; 4 Amen Clinics Inc., Irvine, CA 92626, USA; dkeator@amenclinic.com (D.B.K.); daniel@amenclinic.com (D.A.); 5 Department of Psychiatry and Human Behavior, University of California, Irvine, Irvine, CA 92697, USA; 6 Change Your Brain Change Your Life Foundation, Costa Mesa, CA 92626, USA; 7 Imaging Genetics Center, Mark and Mary Stevens Neuroimaging and Informatics Institute, Keck School of Medicine, University of Southern California, Marina del Rey, CA 90292, USA; pthomp@usc.edu (P.M.T.); neda.jahanshad@usc.edu (N.J.); 8 Department of Psychiatry and Behavioral Health, The Ohio State University College of Medicine, Columbus, OH 43210, USA; jessica.turner@osumc.edu; 9 Clinical Translational Neuroscience Laboratory, Department of Psychiatry and Human Behavior, University of California Irvine, Irvine, CA 92697, USA; tvanerp@hs.uci.edu; 10 Center for the Neurobiology of Learning and Memory, University of California Irvine, 309 Qureshey Research Lab, Irvine, CA 92697, USA; 11 Tri-Institutional Center for Translational Research in Neuroimaging and Data Science, Georgia State University, Georgia Institute of Technology, Emory University, Atlanta, GA 30030, USA; vcalhoun@gsu.edu; 12 Institute of Neuroscience and Medicine, Brain and Behavior (INM-7), Research Centre Jülich, 52428 Jülich, Germany; j.dukart@fz-juelich.de; 13 Institute of Systems Neuroscience, Medical Faculty, University Hospital Düsseldorf, Heinrich Heine University Düsseldorf, 40225 Düsseldorf, Germany

**Keywords:** cerebral blood flow, partial volume correction, prediction, support vector machine, rsfMRI

## Abstract

**Background:** Regional cerebral blood flow (rCBF) is a putative biomarker for neuropsychiatric disorders, including major depressive disorder (MDD). **Methods:** Here, we show that rCBF can be predicted from resting-state functional MRI (rsfMRI) at the voxel level while correcting for partial volume averaging (PVA) artifacts. Cortical patterns of MDD-related CBF differences decoded from rsfMRI using a PVA-corrected approach showed excellent agreement with CBF measured using single-photon emission computed tomography (SPECT) and arterial spin labeling (ASL). A support vector machine algorithm was trained to decode cortical voxel-wise CBF from temporal and power-spectral features of voxel-level rsfMRI time series while accounting for PVA. Three datasets, Amish Connectome Project (*N* = 300; 179 M/121 F, both rsfMRI and ASL data), UK Biobank (*N* = 8396; 3097 M/5319 F, rsfMRI data), and Amen Clinics Inc. datasets (*N* = 372: *N* = 183 M/189 F, SPECT data), were used. **Results:** PVA-corrected CBF values predicted from rsfMRI showed significant correlation with the whole-brain (*r* = 0.54, *p* = 2 × 10^−5^) and 31 out of 34 regional (*r* = 0.33 to 0.59, *p* < 1.1 × 10^−3^) rCBF measures from 3D ASL. PVA-corrected rCBF values showed significant regional deficits in the UKBB MDD group (Cohen’s *d* = −0.30 to −0.56, *p* < 10^−28^), with the strongest effect sizes observed in the frontal and cingulate areas. The regional deficit pattern of MDD-related hypoperfusion showed excellent agreement with CBF deficits observed in the SPECT data (*r* = 0.74, *p* = 4.9 × 10^−7^). Consistent with previous findings, this new method suggests that perfusion signals can be predicted using voxel-wise rsfMRI signals. **Conclusions:** CBF values computed from widely available rsfMRI can be used to study the impact of neuropsychiatric disorders such as MDD on cerebral neurophysiology.

## 1. Introduction

Cerebral blood flow (CBF) is a rigorously maintained physiological parameter responsible for the brain’s metabolic equilibrium and normal neurometabolic function [1]. Regional CBF (rCBF) alterations are associated with aging, cardiometabolic, and neuropsychiatric illnesses, including major depressive disorder (MDD) and others [2,3,4,5,6,7]. CBF takes up ~15% of the cardiovascular output [8], and there is a tight coupling between rCBF and cerebral metabolism metabolic homeostasis [9]. In MDD, single-photon emission computed tomography (SPECT) and positron emission tomography (PET) studies suggest that regional cortical hypoperfusion is a specific biomarker linked to symptoms, cognitive deficits, and clinical outcomes [5,10,11,12,13]. Specifically, lower rCBF in frontal areas and the cingulum can serve as a prognostic tool to infer the successes of therapies, including deep-brain stimulation for treatment-resistant depression [4,5,14]. However, most of the rCBF studies in MDD and other neuropsychiatric disorders to date were performed in samples with modest statistical power, as CBF data are costly and challenging to collect [5]. For example, a recent meta-analysis of 42 studies reported an average sample size of *N* = 42 ± 26 (ranging from 11 to 100), and ~75% of the studies had fewer than 50 participants [14]. This contributes to the high heterogeneity of rCBF findings in MDD and other disorders [14,15,16]. Here, we demonstrate that a practical proxy for rCBF can be derived from resting-state functional MRI (rsfMRI). The advantage of this approach is that rsfMRI data are routinely collected in large and inclusive cohorts, including the major national biobanks and clinical neuroimaging consortia, thus providing statistically powerful samples to quantify regional differences. We demonstrate how findings in predicted rCBF data from a large sample of subjects with MDD and controls shows excellent agreement with ground-truth measures derived from the classical SPECT-CBF measurement approach.

rCBF can be measured non-invasively using nuclear medicine techniques (SPECT/PET) or with arterial spin labeling (ASL) MRI. It is quantified as the blood volume that flows per unit mass per unit time in brain tissue (mL/100 g/min). rCBF data can be captured at rest, during cognitive or behavioral tasks, or during a physiological challenge such as hypercapnia [17]. Existing approaches to quantify rCBF have limitations that have prevented their use in large and inclusive national and international biobanking efforts. SPECT and PET can map CBF using radioactive blood flow tracers but they are limited by high cost per study, radiation safety concerns—especially in healthy participants—and low spatial resolution [18]. ASL imaging uses magnetically labeled blood as an exogenous tracer and calculates CBF maps by fitting a multicompartmental perfusion model using images acquired with and without labeling of inflowing blood [19]. However, ASL studies also suffer from methodological limitations that have prevented their wide use, including a relatively long acquisition time, low signal-to-noise ratio, and variable labeling performance. Together, these challenges have limited most rCBF studies, leading to challenges in replication and reproducibility of biological findings.

Research carried out by our group and others shows that the measures extracted from rsfMRI time series can serve as a proxy for rCBF [20,21]. RsfMRI evaluates the properties of brain function by measuring changes in deoxyhemoglobin associated with neuronal activation in the resting state; this signal also reflects some of the changes underlying CBF [22,23,24,25,26,27,28]. We used a modification of the regional homogeneity quantification approach to derive a proxy for voxel-wise CBF values that shows empirical sensitivity to both metabolic and neuropsychiatric illnesses [2,3]. Chand and colleagues demonstrated that machine learning can be used to predict rCBF from variance in the rsfMRI time series [29]. In their study, they trained a support vector network to predict accuracy for rCBF values using atlas-based measurements of the frequency power spectrum. Their approach showed a good agreement between whole-brain predicted and measured CBF, but the prediction for rCBF was variable by region. The potential issues contributing to this suboptimal prediction may be due to confounding effects of partial volume averaging (PVA)—a situation explained below. The novelties of this study from a methodological perspective include (A) prediction being performed at the voxel level and the information from neighboring voxels also being incorporated to achieve better prediction accuracy, (B) spectral features being extracted from a wide range of frequencies compared to previous studies, and (C) the inclusion of PVA correction. We included three datasets and compared case–control effects between predicted and measured CBF.

The SPECT-, PET-, and MRI-based perfusion imaging approaches typically allow insufficient spatial resolution to resolve the cortical gray matter (GM) ribbon that is evident on high-resolution T_1_-weighted anatomical MRI. This limited resolution leads to PVA artifacts because a single voxel may contain both high-and-low CBF GM and neighboring white matter (WM)/cerebrospinal fluid (CSF) [30]. This PVA of multiple tissues leads to artificially lowered cortical CBF estimates, which are reduced by a fraction dependent on the ratio of the voxel size to the regional GM thickness or, more precisely, the GM volume within the voxel. This is especially important for quantitative rCBF studies in neuropsychiatric illnesses with regionally specific reductions in cortical GM, as it causes artificially lower rCBF values in cases vs. controls [31]. Therefore, quantitative measures of rCBF should account for PVA effects [31,32]. Here, we show that the variable regional performance of the original approach by Chand and colleagues may have been caused by a failure to account for PVA. We expand their model to include partial voxel occupancy values to correct the reduction in rCBF influenced by PVA effects. We also train the original model and show significant changes in the ability to accurately predict rCBF values and regional MDD-related hypoperfusion.

Here, we propose to use the support vector machine (SVM) method to link the information contained in the spectral features extracted from voxel-wise time-series data and voxel-level CBF. Voxel-wise estimates, in contrast to regions-of-interest approaches used before, can possibly correct for partial voxel occupancy and provide a better rCBF proxy independent of the underlying atlas. SVM is a powerful and flexible supervised machine learning method specifically designed for prediction problems with correlated features, such as neighboring voxel-wise time series; the use of different kernel functions in SVM makes it especially effective in handling high-dimensionality and complex non-linear and non-separatable patterns common in voxel-wise imaging data. We aim to demonstrate that the information and insight gained by training the SVM model is translatable across diverse datasets.

## 2. Materials and Methods

### 2.1. Study Sample

**ACP:** We analyzed data from a family-based cohort scanned with MRI as a part of the Amish Connectome Project (ACP), consisting of 300 Amish participants (179 M/121 F, mean age ± s.d.: 37.5 ± 16.3 years) who had both rsfMRI and ASL data available (https://www.humanconnectome.org/study/amish-connectome-project, accessed on 1 June 2025). All participants provided written informed consent on forms approved by the Institutional Review Board of the University of Maryland Baltimore, Baltimore, MD, USA.

**UKBB:** The UKBB sample consisted of 18,898 participants (8833 M/10,065 F, mean age ± s.d.: 63.2 ± 7.5 years) with rsfMRI and volumetric 3D T_1_-weighted MRI data. We used the UKBB parser software to classify participants into subgroups based on their ICD codes, medication information, symptom severity, hospital records, self-reported diagnoses, and other variables using a previously published schema [33]. We separated this sample into MDD cases, *N* = 2290 participants (790 M/1500 F, mean age ± s.d.: 62.1 ± 7.4 years) with recurrent MDD, and *N* = 6106 participants (2287 M/3819 F, mean age ± s.d.: 61.9 ± 7.1 years) who were free of MDD or any other psychiatric condition. The University of Maryland Baltimore provided the initial ethical approval for using the UKBB datasets.

**Amen Clinics Inc.:** The Amen Clinics Inc. (ACI) sample included *N* = 372 participants (*N* = 183 M/189 F, mean age ± s.d.: 45.3 ± 17.1 years). This sample consisted of healthy controls and individuals experiencing symptoms of depression: *N* = 296 patients with recurrent or first-episode MDD (*N* = 183/113 M/F, mean age ± s.d.: 46.1 ± 17.2 years) and *N* = 76 healthy controls (34 M/42 F, mean age ± s.d.: 42.2 ± 17.2 years). All patients in the study gave informed consent to have their anonymous data used in future research at the time of their initial visit to the clinic. The study was approved by the Integ Review Board (004-Amen Clinics Inc.) on 19 September 2014 and determined to be except category 4.

The demographics, inclusion/exclusion criteria, symptom scale ratings, and sample characteristics for all study samples are detailed in the Supplementary Materials.

### 2.2. Arterial Spin Labeling Data Acquisition, Processing, and CBF Extraction

A 3T Siemens Prisma scanner with 64 channels and three-dimensional (3D) pseudo-continuous ASL (pCASL) was used to acquire the arterial spin labeling (ASL) data that included 13 pairs of labeled and control scans (see Supplementary Materials). A 3D T_1_-weighted image was acquired for anatomical reference for tissue segmentation. A brain volume (M_0_) was also acquired without background suppression to normalize the control-label difference for CBF quantification. A standard single-compartment ASL model was used for CBF perfusion estimation, and partial volume effects correction was performed with a spatially regularized method [34] using the FSL software package. Spatial regularization, motion correction, and partial volume corrections were performed in FSL v6.0.1. The high-resolution structural image provided partial volume estimates (PVEs) for the different tissue types (gray matter (GM), white matter (WM), and cerebrospinal fluid (CSF)). Partial volume-corrected CBF maps were used to extract the quantitative voxel-wise CBF signals using the volumetric Desikan–Killiany (DK) atlas that consisted of thirty-four cortical brain regions from each hemisphere.

### 2.3. RsfMRI Data Acquisition, Processing, Time-Series Extraction, and Spectral Features

For ACP participants, there were two rsfMRI scans; oblique axial acquisitions alternated between phase encoding in the anterior-to-posterior (AP) and posterior-to-anterior (PA) directions within a single run. Separate single-band reference images, acquired similarly to resting scans, were used for spatial distortion correction. UKBB rsfMRI data were acquired on 3T Siemens Skyra scanners with the standard Siemens 32-channel receive head coil (full protocols in the Supplementary Materials).

The rsfMRI data were processed using the analysis workflow developed by the Enhancing Neuro Imaging Genetics through Meta-Analysis (ENIGMA) consortium; details of the processing steps have been described in prior publications [35,36] and the Supplementary Materials. The preprocessed data were then used to extract the voxel-wise time-series data using the volumetric DK atlas, which consisted of thirty-four cortical brain regions from each hemisphere. Voxel-wise time-series data were used to calculate the voxel-wise spectral features from the power spectrum using Fourier transformation, and the spectral features were then used in the rCBF prediction analysis.

### 2.4. Structural MRI Data Collection and Partial Voxel Occupancy Calculation

ACP T_1_-weighted imaging. High-resolution (0.8 mm isotropic), high-GM-WM contrast (~25%) T_1_-weighted images were acquired using a retrospective motion-corrected protocol [37] using a 3T Siemens Prisma scanner equipped with a 64-channel head coil (see the Supplementary Materials for details). Processing of T_1_-weighted structural data was performed using the HCP preprocessing pipeline [38]. Briefly, we used T_1_-weighted images produced by the PreFreeSurfer part of the workflow: correction for shape distortions, B_1_ homogeneity correction, registration to rsfMRI images for the same subject, registration to the MNI space, and removal of the non-brain tissue.

UKBB T_1_-weighted imaging. Three-dimensional T_1_-weighted imaging (data field 20252) was performed at an isotropic resolution of 1 mm using a 3T Siemens Skyra scanner equipped with a 32-channel coil. We used T_1_-weighted images following registration to the MNI space, correction of B_1_ homogeneity, and removal of the non-brain tissue.

Following this preprocessing, T_1_-weighted ACP and UKBB images were segmented into GM, WM, and CSF using the *FSL-FAST* tool v6.0.1, which provided partial tissue segmentation maps [39]. The GM partial occupancy maps were smoothed with a 5 mm full width at half maximum Gaussian filter and resampled to 2 mm isotropic resolution to match the rsfMRI data. The partial voxel occupancy maps for GM were used to train the PVA-corrected CBF predictor.

### 2.5. SPECT Data Acquisition, Processing, and Analyses

SPECT scans were acquired using a Picker (Philips) Prism XP 3000 triple-headed gamma camera (Picker Int. Inc., Ohio Nuclear Medicine Division, Bedford Hills, OH, USA) with low-energy, high-resolution fan beam collimators. For each procedure, a weight-appropriate dose of 99 mTc–hexamethylpropyleneamine oxime was administered intravenously at rest while the subjects sat in a dimly lit room with their eyes open. Subjects were scanned for approximately 30 min after injection. Data acquisition yielded 120 images per scan, with each image separated by three degrees, spanning 360°. A low-pass filter was applied with a high cutoff, and Chang attenuation correction was performed [40]. The final reconstructed image was 128 × 128 × 78, with a voxel size of 2.5 mm isotropic.

### 2.6. CBF Prediction Based on Voxel-Wise Cortical rsfMRI Data

**Figure 1:**
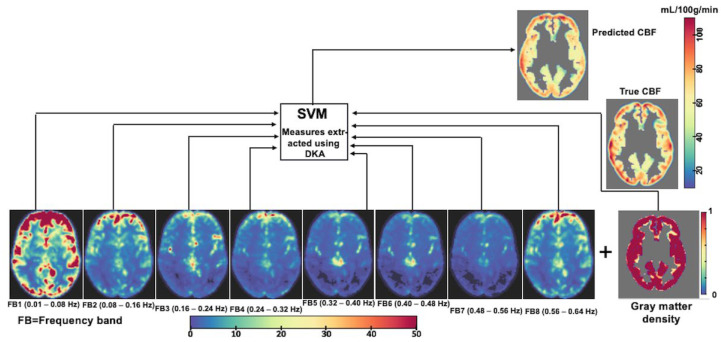
CBF prediction scheme using voxel-wise cortical rsfMRI data (sample for a representative subject). R package e1071 provided the functionality for the support vector model (SVM) regression, and the radial basis function kernel was used. We used an eight-bin schema of the spectral power density of the voxel-wise rsfMRI time series, FB1 to FB8 (FB: frequency band), and frequency range values for each band were provided. In addition, a value between 0 and 1 for gray matter (GM) tissue occupancy was used as an additional feature for the SVM model.

The R package “e1071” [41] provided functionality for the support vector model (SVM) regression with a radial basis function kernel [42]. We used the eight-bin schema of spectral power density of the voxel-wise rsfMRI time series (with midpoints of 0.04, 0.12, 0.20, 0.28, 0.36, 0.44, 0.52, and 0.60 Hz). This range of frequencies covered the frequency range of the signals captured in our fMRI acquisition, the ACP dataset. These frequency ranges included the frequency bands reported by Chand and colleagues in their study [29]. This created a feature vector of eight elements per voxel. We limited this analysis to cortical voxels (based on the population-wide template) to remove the subcortical signals (Figure 1). We combined the features from six nearest neighboring voxels to improve the SNR (in total, 56 spectral features per voxel). In addition, a value between 0 and 1 (1 indicating 100%) of GM tissue occupancy was used as an additional feature for the SVM model. During the preliminary stages, several prediction models were explored, including Random Forest and Gradient Boost approaches. However, SVM emerged as the better performer compared to the other methods. The choices of the two most important tunning parameters in SVM, “gamma” and “C”, were determined based on 10-fold cross-validation results. In addition, we split our data into a training set and a testing set. We trained the model using two approaches: with and without partial occupancy. For the latter, only the spectral features were used for prediction. We identified an optimal SVM model from the training set and evaluated the prediction accuracy (observed vs. predicted CBF correlation) of the selected model in the testing set. The prediction accuracy of our model in the testing dataset was reasonably close to the prediction in the training dataset. The model was trained twice. At first, the partial occupancy parameter was set to 0 for all voxels, which corresponded to the model used by Chand and colleagues [29]. Next, the partial GM occupancy parameter was provided from the partial voxel segmentation map of the corresponding T_1_-weighted image; the values ranged from 0 (no GM occupancy) to 1 (full GM occupancy). The computation work for the prediction of CBF signals, including the partial occupancy metrics in the analysis, was carried out for each voxel, voxel by voxel, instead of using the average signal from the given brain regions, as in the study by Chand and colleagues [29]. However, all the results, finally, were derived by averaging all the voxel-level computed measures across the sixty-four DK atlas regions.

### 2.7. Analysis

All statistical analyses were performed in R v4.3.2. Analyses were focused on evaluation of PVA correction (including the partial occupancy metrics) for predicting voxel-wise CBF from voxel-wise rsfMRI data and were performed in two independent cohorts. In the ACP cohort, where both rsfMRI and CBF data were available, we evaluated the effects of PVA correction on the accuracy of the CBF prediction. The ACP dataset was randomly split, with 2/3 of the data in the training set and 1/3 in the testing set. Note that the split was performed in such a way that there was no age or sex difference between them, and diagnosis was not taken into consideration. The testing dataset was used to perform a correlation analysis of predicted and actual CBF for whole-brain average and 34 regional CBF values calculated using the DK atlas regions. We used Bonferroni correction for multiple comparisons (*N* = 34).

In UKBB, only rsfMRI data were available. We used PVA-corrected CBF prediction to evaluate the pattern of regional CBF differences between MDD individuals and healthy controls (HCs). We used Student’s *t*-tests to compare the rCBF, as predicted from rsfMRI for MDD vs. HC group differences, and calculated *Cohen’s d* effect sizes, and their significance was determined based on multiple comparison correction, *N* = 34.

In the Amen Clinics Inc. sample, only SPECT-measured CBF was available. We processed the data, extracted the regional CBF values using DK atlas regions and then calculated regional effect sizes for rCBF differences between MDD cases and HCs for each of these atlas regions. Finally, the regional effect sizes for the rCBF differences between MDD cases and HCs computed based on the rsfMRI data were compared to the corresponding regional effect sizes obtained from the SPECT data. All the codes for CBF prediction, statistical analyses, and examples are available at https://github.com/kehongjie/rsfMRI_CBF, accessed on 1 June 2025.

## 3. Results

### 3.1. Effects of Including PVA on rCBF Prediction in the ACP Sample

The model parameter training step was performed using ~2 × 10^5^ voxels comprising the cortical GM for each subject in the training dataset. The prediction accuracy of the original (uncorrected) and partial volume averaging (PVA)-corrected models evaluated in the testing dataset are shown in Table 1. True, PVA-corrected/uncorrected CBF maps, computed using the voxel-wise rsfMRI time-series data with and without PVA correction, from a representative subject are shown in Figure S2. The PVA-corrected CBF values averaged for the whole brain showed numerically higher correlation with measured, whole-brain CBF values (*r* = 0.68 vs. 0.50, *p* = 2.5 × 10^−13^ vs. 4.2 × 10^−7^, for corrected vs. original), and the difference in correlation coefficients approached statistical significance (*Z* = 1.95, *p* = 0.05).

The predicted and measured voxel-wise CBF values were averaged in the testing dataset for the *N* = 34 DK atlas cortical regions that provided the regional metrics. The uncorrected (original) model showed no significant correlations between measured and predicted CBF values (for *N* = 34 multiple-comparison correction). In contrast, the PVA-corrected model showed significant correlation between predicted and measured CBF for 31 regions (average *r* ± s.e. = 0.43 ± 0.07). This improvement was statistically significant (*p* = 3.0 × 10^−5^) based on a pairwise t-test when compared to the average correlation coefficient observed for the original model (average *r* ± s.e. = 0.14 ± 0.04). The CBF values predicted by the PVA-corrected model were significantly correlated with measured CBF values for 31 out of 34 regions (*r* = 0.32 to 0.59, all *p* < 0.0015). The correction was nominally significant (*p* < 0.05) for the remaining three structures: pars triangularis (PTR, *r* = 0.29), rostral middle frontal gyrus (RMFG, *r* = 0.31), and transverse temporal gyrus (TTG, *r* = 0.27). The difference in the correlation coefficients between the original and PVA-corrected models was significant (*p* < 0.05) for 18 regions (Table 1).

### 3.2. rCBF Differences Between the UKBB MDD Cohort and the Amen Clinics Inc. Cohort

**Figure 2:**
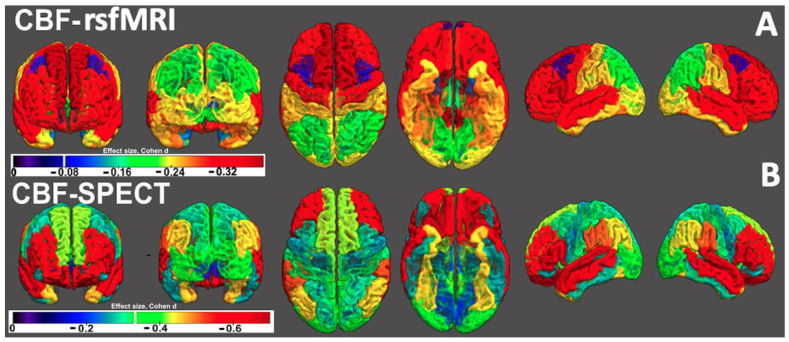
Brain surface rendered effect size maps: The effect sizes of the (**A**) predicted CBF (for corrected model) differences between MDD cases and controls for the UKBB cohort and (**B**) CBF differences between MDD cases and controls in the Amen Clinic Inc. cohort. Each subplot was supplied with a color bar, and a vertical white mark in the color bar represents the color that corresponds to corrected *p* < 0.05 (multiple corrections). Note that the stronger negative effect sizes are represented by hotter colors.

**Figure 3:**
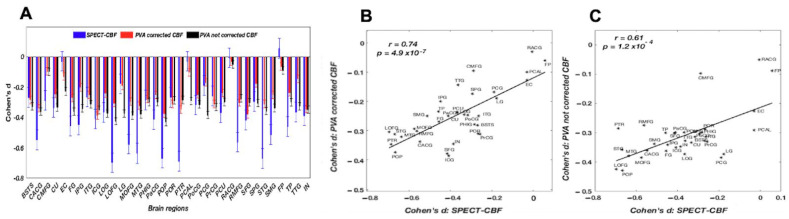
*Cohen’s d* effect sizes for predicted CBF with and without partial voxel averaging (PVA) correction and CBF measured by SPECT. Subplot (**A**) shows regional effect size values, whereas subplots (**B**,**C**) show the relationship between MDD-related regional CBF differences as measured by SPECT and the MDD-related regional differences based on rsfMRI-derived CBF measurement with and without PVA correction.

The voxel-wise PVA-corrected CBF values for the UKBB subjects were averaged to produce 34 regional rCBF values. The effect sizes of the predicted CBF (for corrected model) differences between MDD cases and HCs are shown in Figure 2 and Figure 3A. The stronger effect sizes were more negative and are represented by hotter colors. MDD was associated with significantly lower CBF for 32 cortical regions. The strongest negative effect sizes were observed in the superior frontal, inferior parietal, and postcentral gyri (effect sizes: −0.38/−0.37 ± 0.2, *p* < 10^−16^). The predicted CBF differences for fusiform and rostral anterior cingulate areas did not show significant differences between participants with MDD and HCs. The PVA-corrected rCBF values in the UKBB controls showed a strong positive correlation with the PVA-corrected rCBF values in the ACP cohort (Figure S3).

The rCBF effect sizes in the Amen Clinics Inc. cohort showed regions where MDD cases differed significantly from HCs, and these were significant for 18 out of 34 regions (*d* = −0.70 to −0.36, *p* < 0.0015; *d* means *Cohen’s d* in this manuscript) (Table 2). The highest regional effect sizes were observed for the lateral orbitofrontal (LG) (*d* = −0.70) followed by the precuneus (PCU) and supramarginal (SG) areas (*d* = −0.67) (see Figure 3A).

The regional effects for predicted CBF values in the UKBB cohort showed statistically significant positive correlations with those computed from the Amen Clinics Inc. SPECT data (*r* = 0.74, *p* = 4.9 × 10^−7^ (see Figure 3B). This suggests a good agreement between the MDD-related rCBF differences as measured by SPECT and the MDD-related regional differences based on rsfMRI-derived CBF measurement.

In the post hoc analyses, we re-calculated the CBF effect sizes in the UKBB sample using the original, non-PVA-corrected model by Chand and colleagues (Table S1). The computed effect sizes were also significantly correlated with the effect sizes observed from the Amen Clinics Inc. cohort, but the correlation coefficient was numerically lower (Figure 3C). We further calculated the MDD-related effect sizes for regional cortical thickness in the UKBB sample (Table S2). These effect sizes were not significantly correlated with predicted CBF values (*p* > 0.4). However, effect sizes for cortical GM thickness in the UKBB were significantly correlated with those reported by the ENIGMA-MDD workgroup (*r* = 0.64, *p* = 5 × 10^−4^). Moreover, the differences in the numbers of voxels that the ROIs contained showed no differences in predicted and measured voxel-wise CBF values between groups and datasets. We further calculated and tabulated correlation coefficients with age for the rCBF values predicted using the original and PVA-corrected rCBF values for the non-psychiatric controls of the UKBB sample (Table S3). The correlation coefficients between regional measurements of the cortical GM thickness and age were also calculated. The scatter plots between regional *r*-values for rCBF and GM thickness showed that the regional variance in rCBF values predicted using the original model was significantly explained by a reduction in cortical GM thickness with age (*r* = 0.43, *p* = 0.01) (Figure S4A). The PVA-corrected rCBF showed no significant correlation with aging-related trends in cortical GM thickness (*r* = 0.11, *p* = 0.54) (Figure S4B).

## 4. Discussion

This study demonstrated that a proxy measurement for rCBF, a sensitive phenotype for neuropsychiatric and metabolic illnesses, can be derived from widely available rsfMRI. We extended the original work by Chand and colleagues which found that the spectral features of rsfMRI signals [29] can be used to decode rCBF. The study by Chand and colleagues was performed using averaged region-wise data and showed variable, by region, performance in predicting rCBF. We performed this analysis at the voxel-wise level, while correcting for PVA artifacts. This improved the ability of the original method to accurately predict rCBF. We found that rCBF values, predicted from rsfMRI data, demonstrate a pattern of significant effect sizes associated with the recurrent MDD in a large and inclusive sample of participants in the UKBB study. This regional pattern showed good agreement with the pattern of rCBF differences in MDD patients measured using the SPECT modality. Overall, we showed that a useful and practical rCBF proxy signal can reliably be predicted from the rsfMRI data, which are widely available in many large-scale biobanks and clinical cohorts with neuroimaging, thus providing a valuable phenotype for studies of neuropsychiatric disorders where more direct measures of rCBF may not be available.

The study by Chand and colleagues used the SVM to decode rCBF signals from the spectral features extracted from region-wise average rsfMRI signals. We made this approach more focused and practical by performing this prediction on a voxel-by-voxel level. This offers two advantages over region-of-interest approaches: the voxel-wise prediction can be corrected for PVA, and the predicted rCBF signal is independent of the underlying atlas/regions of interest. The PVA correction is necessary for quantitative analysis of the nuclear medicine studies, including SPECT and PET, where the sampling resolution (voxel dimensions of 4–8 mm) is insufficient to resolve the cortical GM ribbon [31,32]. The rsfMRI and ASL sequences have a much higher spatial resolution (~2–3 mm) than PET or SPECT data. However, PVA is still needed for quantitative rCBF analysis in studies of neuropsychiatric disorders, where the lower GM thickness in cases can lead to an artificial apparent reduction in the measured rCBF. The scatter plots between regional *r*-values for rCBF and GM thickness showed that the regional variance in rCBF values predicted using the original model was significantly explained by a reduction in cortical GM thickness with age (Figure S4A). The PVA-corrected rCBF showed no significant correlation with aging-related trends, and this was also true for cortical GM thickness (Figure S4B). These finding replicate previous reports by our group and others that uncorrected, average cortical uptake values are proportional to regional cortical thickness values and that the aging trends in PVA-corrected cortical uptake values are independent of the aging trends in cortical thickness [31,32,43,44].

We used CBF difference in MDD to demonstrate the practical application of rCBF prediction. The PVA-corrected rCBF predictions from rsfMRI showed remarkable agreement with rCBF patterns derived using the classical SPECT approach from an independent clinical sample of people experiencing an acute depressive episode. We chose MDD as it is one of the most common neuropsychiatric illnesses affecting ~10% of the population. Findings of cerebral hypoperfusion in MDD are replicable and predictive of the clinical state and treatment outcome [4,5,43]. On the other hand, subjects with MDD show only modest differences in cortical GM thickness, which is, likewise, a replicable finding [42,43]. Research findings from the SPECT/PET data were used to hypothesize that MDD is specifically associated with the hypoperfusion of the limbic–frontal circuitry, including the dorsolateral frontal and cingulate areas, and linked it to cognitive and behavioral deficits [45]. This consistency in regional findings between rsfMRI measures (local connectivity) and CBF provides a plausible physiological mechanism by linking patterns of lower regional values to patterns of lower regional CBF. Lower rsfMRI values in the cingulate, frontal, and temporal areas were reported in patients with MDD and were correlated with symptom severity and cognitive performance [46,47]. These areas show the highest MDD-related effect sizes in the UKBB and Amen Clinics Inc. datasets. However, the effect sizes were statistically significant; they were mostly small to medium, and those that included parietal and temporal areas were more widespread. The UKBB sample consisted of 8396 participants, a comparatively larger sample size, with a mean age ± s.d. of 62.0 ± 7.2 years, which afforded higher statistical power (significant rCBF reductions in 32/34 regions), whereas the ACI sample consisted of 372 participants, a comparatively smaller sample size, with a mean age ± s.d. of 45.3 ± 17.1 years, thus leading to larger variation in the effect sizes (significant rCBF reductions in 18/34 regions). Note that the UKBB MDD participants were a non-treatment-seeking population, whereas the ACI participants were specialty treatment-seeking MDD patients. Our post hoc analysis demonstrated that the pattern of regional effects for the PVA-corrected rCBF pattern did not overlap with the effects of the MDD on regional cortical thickness, yet the MDD effect on cortical thickness was in good agreement with those reported by ENIGMA-MDD workgroup.

This study has limitations. The original prediction approach piloted by Chand and colleagues was focused on cortical phenotypes and excluded the subcortical brain structures. We likewise limited our analysis to the cortical voxels and excluded subcortical regions. The rCBF prediction and PVA correction for the subcortical regions will likely require training of a separate SVM predictor (which needs to be explored) because the patterns of association between power spectra and CBF differ between cortical and subcortical regions. Likewise, the patterns of PVA may differ as well because subcortical GM nuclei often include penetrating white matter tracts, where accurate GM/WM ratios will be needed, thus requiring a different PVA correction training. Moreover, including the contribution of WM CBF and WM voxel occupancy measures for predicting CBF values remains to be considered and explored in detail in a future study. In addition, it is unlikely that the outcomes of the training will be translatable across all rsfMRI protocols. We showed that the training of the rCBF predictor on the ACP data showed good performance in the UKBB sample, but both datasets were collected using Siemens 3T scanners and multiband sequences with similar acquisition parameters. Significant deviation from this protocol is expected to require a re-training of the rCBF prediction approach. Of the three datasets, only the ACP dataset includes both ASL and rsfMRI data from the same subjects, allowing for direct validation of rsfMRI-predicted CBF against ASL measurements, but the UKBB dataset provides only rsfMRI data, while ACI includes only SPECT data. The validation of these datasets was indirect, relying on effect size correlations. Moreover, our analysis included possible frequency ranges in rsfMRI data, which include respiratory cycles and frequency (>0.5 Hz). We found no association between the spectral power density and noise calculated from raw rsfMRI data (Figure S5). The effects of the physiological noise components could have been corrected if physiological recordings for respiratory and cardiac cycles were available, which would have strengthened the spectral features’ robustness. In addition, this study mainly aimed to determine the overall trend of contrast between MDD and control subjects at the population level; how it differs from other clinical characteristics needs to be explored in future studies or clinical trials specifically focusing on MDD patients, with MDD subtypes taken into consideration. Note that while age and sex differ across the cohorts, inferences drawn here still need to be interpreted with caution, even though the regional effects for predicted CBF from rsfMRI data in the UKBB sample were significantly correlated with the CBF measured with the SPECT data in the Amen Clinics Inc. sample. We here used the machine learning approach that captures statistical associations rather than physiologically meaningful relationships. It still opens up the avenue for using a biophysical model as an alternative approach that strengthens the interpretability and reliability of research findings needed for clinical translation.

## 5. Conclusions

This study demonstrated that rCBF can be predicted from the frequency spectra of rsfMRI data. This provides an opportunity to study effects of neuropsychiatric illnesses on rCBF because of the much wider availability of rsfMRI compared to ASL/SPECT/PET data in large and inclusive datasets such as the UKBB, ABCD, and HCP datasets, as well as in the ENIGMA Consortium clinical working groups. We further demonstrated that the correction for PVA is a necessary step during this analysis. PVA-corrected rCBF values showed better agreement with ASL-derived CBF values in the testing dataset and SPECT-derived MDD effect sizes. The uncorrected rCBF values showed statistically weaker association with ASL-derived CBF values and SPECT-derived MDD effect sizes. Overall, a useful and practical rCBF proxy signal can reliably be predicted from the rsfMRI data, which can be a putative biomarker for brain disorders.

## Figures and Tables

**Table 1:** Regional correlation coefficients between predicted rCBF values (original and PVA-corrected) and rCBF values measured using ASL during the same session (ACP cohort). The statistically significant values after multiple comparison corrections are bolded (*p* < 0.05/34 = 1.5 × 10^−3^). The differences between the correlation coefficients (z-value) are presented.

Name of Region	Abbreviation	Original Model	Corrected Model	Z-Value of Difference (Significance)
Banks of superior temporal sulcus	BSTS	0.26 (*p* = 1.2 × 10^−2^)	**0.42 (*p* = 3.2 × 10^−5^)**	1.21 (*p* = 1.9 × 10^−1^)
Caudal anterior cingulate	CACG	0.16 (*p* = 1.1 × 10^−1^)	**0.38 (*p* = 2.0 × 10^−4^)**	1.64 (*p* = 1.0 × 10^−1^)
Caudal middle frontal gyrus	CMFG	0.13 (*p* = 1.7 × 10^−1^)	**0.41 (*p* = 4.6 × 10^−5^)**	2.11 (*p* = 4.3 × 10^−2^)
Cuneus	CU	0.16 (*p* = 1.2 × 10^−1^)	**0.46 (*p* = 2.7 × 10^−6^)**	2.38 (*p* = 2.3 × 10^−2^)
Entorhinal cortex	EC	0.07 (*p* = 3.2 × 10^−1^)	**0.59 (*p* = 2.8 × 10^−10^)**	4.22 (*p* = 5.4 × 10^−5^)
Fusiform gyrus	FG	0.13 (*p* = 1.8 × 10^−1^)	**0.58 (*p* = 7.4 × 10^−10^)**	3.70 (*p* = 4.3 × 10^−4^)
Inferior parietal gyrus	IPG	0.24 (*p* = 2.0 × 10^−2^)	**0.36 (*p* = 3.4 × 10^−4^)**	0.95 (*p* = 2.5 × 10^−1^)
Inferior temporal gyrus	ITG	0.14 (*p* = 1.5 × 10^−1^)	**0.49 (*p* = 5.2 × 10^−7^)**	2.74 (*p* = 9.3 × 10^−3^)
Isthmus cingulate gyrus	ICG	0.17 (*p* = 8.8 × 10^−2^)	**0.43 (*p* = 1.7 × 10^−5^)**	1.97 (*p* = 5.7 × 10^−2^)
Lateral occipital gyrus	LOG	0.16 (*p* = 1.0 × 10^−1^)	**0.45 (*p* = 5.2 × 10^−6^)**	2.23 (*p* = 3.3 × 10^−2^)
Lateral orbito-frontal gyrus	LOFG	0.09 (*p* = 2.7 × 10^−1^)	**0.49 (*p* = 5.8 × 10^−7^)**	3.10 (*p* = 3.3 × 10^−3^)
Lingual gyrus	LG	0.11 (*p* = 2.1 × 10^−1^)	**0.51 (*p* = 1.4 × 10^−7^)**	3.13 (*p* = 3.0 × 10^−3^)
Medial orbito-frontal gyrus	MOFG	0.09 (*p* = 2.6 × 10^−1^)	**0.42 (*p* = 2.3 × 10^−5^)**	2.50 (*p* = 1.8 × 10^−2^)
Middle temporal gyrus	MTG	0.16 (*p* = 1.1 × 10^−1^)	**0.34 (*p* = 8.5 × 10^−4^)**	1.35 (*p* = 1.6 × 10^−1^)
Para hippocampal gyrus	PHIG	0.09 (*p* = 2.6 × 10^−1^)	**0.55 (*p* = 5.2 × 10^−9^)**	3.70 (*p* = 4.3 × 10^−4^)
Para central gyrus	PaCG	0.07 (*p* = 3.0 × 10^−1^)	**0.49 (*p* = 4.7 × 10^−7^)**	3.23 (*p* = 2.2 × 10^−3^)
Pars-opercularis	POP	0.19 (*p* = 6.8 × 10^−2^)	**0.38 (*p* = 2.1 × 10^−4^)**	1.43 (*p* = 1.4 × 10^−1^)
Pars-orbitalis	POR	0.10 (*p* = 2.3 × 10^−1^)	**0.33 (*p* = 1.1 × 10^−3^)**	1.70 (*p* = 9.5 × 10^−2^)
Pars-triangularis	PTR	0.12 (*p* = 2.0 × 10^−1^)	**0.29 (*p* = 4.7 × 10^−3^)**	1.28 (*p* = 1.8 × 10^−1^)
Pericalcarine	PCAL	0.16 (*p* = 1.2 × 10^−1^)	**0.48 (*p* = 9.1 × 10^−7^)**	2.55 (*p* = 1.5 × 10^−2^)
Postcentral gyrus	PoCG	0.09 (*p* = 2.6 × 10^−1^)	**0.42 (*p* = 2.3 × 10^−5^)**	2.51 (*p* = 1.7 × 10^−2^)
Posterior cingulate gyrus	PCG	0.18 (*p* = 8.0 × 10^−2^)	**0.39 (*p* = 1.0 × 10^−4^)**	1.62 (*p* = 1.0 × 10^−1^)
Precentral gyrus	PrCG	0.09 (*p* = 2.7 × 10^−1^)	**0.46 (*p* = 2.9 × 10^−6^)**	2.86 (*p* = 6.7 × 10^−3^)
Precuneus	PCU	0.18 (*p* = 7.7 × 10^−2^)	**0.42 (*p* = 2.6 × 10^−5^)**	1.84 (*p* = 7.0 × 10^−2^)
Rostral anterior cingulate gyrus	RACG	0.17 (*p* = 9.6 × 10^−2^)	**0.36 (*p* = 3.9 × 10^−4^)**	1.45 (*p* = 1.4 × 10^−1^)
Rostral middle frontal gyrus	RMFG	0.09 (*p* = 2.7 × 10^−1^)	0.31 (*p* = 2.6 × 10^−3^)	1.62 (*p* = 1.1 × 10^−1^)
Superior frontal gyrus	SFG	0.09 (*p* = 2.8 × 10^−1^)	**0.45 (*p* = 5.0 × 10^−6^)**	2.79 (*p* = 8.1 × 10^−3^)
Superior parietal gyrus	SPG	0.17 (*p* = 9.8 × 10^−2^)	**0.49 (*p* = 6.6 × 10^−7^)**	2.51 (*p* = 1.7 × 10^−2^)
Superior temporal gyrus	STG	0.15 (*p* = 1.3 × 10^−1^)	**0.35 (*p* = 5.7 × 10^−4^)**	1.50 (*p* = 1.3 × 10^−1^)
Supramarginal gyrus	SMG	0.23 (*p* = 2.8 × 10^−2^)	**0.40 (*p* = 7.4 × 10^−5^)**	1.33 (*p* = 1.7 × 10^−1^)
Frontal pole	FP	0.12 (*p* = 1.9 × 10^−1^)	**0.53 (*p* = 3.6 × 10^−8^)**	3.23 (*p* = 2.1 × 10^−3^)
Temporal pole	TP	0.15 (*p* = 1.4 × 10^−1^)	**0.50 (*p* = 2.5 × 10^−7^)**	2.79 (*p* = 8.0 × 10^−3^)
Transverse temporal gyrus	TTG	0.18 (*p* = 7.2 × 10^−2^)	0.27 (*p* = 8.7 × 10^−3^)	0.65 (*p* = 3.2 × 10^−1^)
Insula	IN	0.18 (*p* = 7.7 × 10^−2^)	**0.42 (*p* = 2.3 × 10^−5^)**	1.87 (*p* = 7.0 × 10^−2^)

**Table 2:** Regional MDD effect sizes for predicted rCBF values with PVA correction (UKBB cohort) and for rCBF values measured using SPECT (Amen Clinics Inc. cohort). The statistically significant values after multiple-comparison corrections are in boldface (*p* < 0.05/34 = 1.5 × 10^−3^).

Region	Abbreviation	MDD Effect Size (UKBB)	MDD Effect Size (Amen Clinics Inc.)
Banks of superior temporal sulcus	BSTS	**−0.28 (*p* < 10^−16^)**	−0.27 (*p* = 0.01)
Caudal anterior cingulate	CACG	**−0.34 (*p* < 10^−16^)**	**−0.55 (*p* = 6 × 10^−7^)**
Caudal middle frontal gyrus	CMFG	**−0.10 (*p* = 4 × 10^−5^)**	−0.29 (*p* = 0.009)
Cuneus	CU	**−0.24 (*p* < 10^−16^)**	−0.33 (*p* = 0.002)
Entorhinal cortex	EC	**−0.13 (*p* = 3 × 10^−8^)**	−0.03 (*p* = 0.4)
Fusiform gyrus	FG	**−0.27 (*p* < 10^−16^)**	**−0.46 (*p* = 3 × 10^−5^)**
Inferior parietal gyrus	IPG	**−0.20 (*p* < 10^−16^)**	**−0.45 (*p* = 3 × 10^−5^)**
Inferior temporal gyrus	ITG	**−0.25 (*p* < 10^−16^)**	−0.27 (*p* = 0.02)
Isthmus cingulate gyrus	ICG	**−0.39 (*p* < 10^−16^)**	**−0.41 (*p* = 2 × 10^−4^)**
Lateral occipital gyrus	LOG	**−0.24 (*p* < 10^−16^)**	**−0.37 (*p* = 1 × 10^−3^)**
Lateral orbito-frontal gyrus	LOFG	**−0.30 (*p* < 10^−16^)**	**−0.70 (*p* = 2 × 10^−10^)**
Lingual gyrus	LG	**−0.19 (*p* = 10^−16^)**	−0.18 (*p* = 0.09)
Medial orbito-frontal gyrus	MOFG	**−0.29 (*p* < 10^−16^)**	**−0.58 (*p* = 1 × 10^−7^)**
Middle temporal gyrus	MTG	**−0.32 (*p* < 10^−16^)**	**−0.64 (*p* = 6 × 10^−9^)**
Para hippocampal gyrus	PHIG	**−0.28 (*p* < 10^−16^)**	−0.28 (*p* = 0.01)
Para central gyrus	PaCG	**−0.25 (*p* < 10^−16^)**	**−0.41 (*p* = 2 × 10^−4^)**
Pars-opercularis	POP	**−0.37 (*p* < 10^−16^)**	**−0.67 (*p* = 1 × 10^−9^)**
Pars-orbitalis	POR	**−0.31 (*p* < 10^−16^)**	−0.27 (*p* = 0.02)
Pars-triangularis	PTR	**−0.35 (*p* < 10^−16^)**	**−0.69 (*p* = 4 × 10^−10^)**
Pericalcarine	PCAL	**−0.10 (*p* = 1 × 10^−5^)**	−0.03 (*p* = 0.4)
Postcentral gyrus	PoCG	**−0.25 (*p* < 10^−16^)**	−0.32 (*p* = 0.004)
Posterior cingulate gyrus	PCG	**−0.17 (*p* = 1 × 10^−14^)**	0.19 (*p* = 0.08)
Precentral gyrus	PrCG	**−0.31 (*p* < 10^−16^)**	−0.26 (*p* = 0.02)
Precuneus	PCU	**−0.24 (*p* < 10^−16^)**	**−0.37 (*p* = 8 × 10^−4^)**
Rostral anterior cingulate gyrus	RACG	−0.03 (*p* = 0.2)	−0.005 (*p* = 0.4)
Rostral middle frontal gyrus	RMFG	**−0.30 (*p* < 10^−16^)**	**−0.56 (*p* = 3 × 10^−7^)**
Superior frontal gyrus	SFG	**−0.38 (*p* < 10^−16^)**	**−0.42 (*p* = 2 × 10^−4^)**
Superior parietal gyrus	SPG	**−0.17 (*p* = 3 × 10^−14^)**	−0.29 (*p* = 0.008)
Superior temporal gyrus	STG	**−0.31 (*p* < 10^−16^)**	**−0.67 (*p* = 1 × 10^−9^)**
Supramarginal gyrus	SMG	**−0.25 (*p* < 10^−16^)**	**−0.51 (*p* = 2 × 10^−6^)**
Frontal pole	FP	−0.06 (*p* = 0.008)	0.06 (*p* = 0.3)
Temporal pole	TP	**−0.24 (*p* < 10^−16^)**	**−0.46 (*p* = 3 × 10^−5^)**
Transverse temporal gyrus	TTG	**−0.14 (*p* = 1 × 10^−8^)**	**−0.36 (*p* = 1 × 10^−3^)**
Insula	IN	**−0.34 (*p* < 10^−16^)**	**−0.39 (*p* = 4 × 10^−4^)**

## Data Availability

Relevant data are made available in the manuscript. Imaging data will be made available upon request. All the codes for CBF prediction, statistical analyses, and examples are available at https://github.com/kehongjie/rsfMRI_CBF (access on 1 June 2025).

## Supplementary Materials

The following supporting information can be downloaded at https://www.mdpi.com/article/10.3390/brainsci15090908/s1: Table S1. Regional MDD effect size for predicted rCBF values with no PVA-correction for the UKBB cohort. Table S2. Correlation between cortical thickness with age and MDD effect size for cortical thickness (UKBB cohort). Table S3. Correlation coefficients between predicted CBF (no PVA-correction and with PVA-correction) with age (UKBB cohort). Figure S1: Brief summary outline illustrating the three datasets used in the study. Figure S2: Representative CBF maps for a participant from the ACP cohort. Figure S3: Relationship between average PVA-corrected regional CBF between the ACP and UKBB cohorts (controls). Voxel-wise CBF values were predicted using voxel-wise rsfMRI timeseries data first and then signals were averaged from corresponding voxels to get the average regional CBF values. Figure S4: Relationship between the correlation values for gray matter (GM) thickness vs. age and the correlation values for predicted regional CBF values (no PVA-corrected and PVA-corrected) vs. age. Figure S5: Relationship between noise signals from the raw rsfMRI data using MPPCA denoising technique and the corresponding band-wise spectral power density measures (*N* = 40). Figure S6: Association between the average predicted rCBF values (DK atlas) from the UKBB healthy controls and the correspondingly measured rCBF values from the Amen Clinics Inc. healthy controls.
