# Supplementary material for: Predicting Regional Cerebral Blood Flow Using Voxel-Wise Resting-State Functional MRI
Source: Brain Sci. 2025 Aug 23;15(9):908. doi: 10.3390/brainsci15090908 (PMC12467352; doi:10.3390/brainsci15090908)
Supplement: Supplementary file 1 [file brainsci-15-00908-s001.zip › brainsci-3760800-SI.pdf]

Supplementary Materials

Predicting cerebral blood flow using voxel-wise resting-state functional MRI

Materials and Methods

Tables

Table S1. Regional MDD effect size for predicted rCBF values with no PVA-correction for the UKBB cohort.

Table S2. Correlation between cortical thickness with age and MDD effect size for cortical thickness (UKBB cohort).

Table S3. Correlation coefficients between predicted CBF (no PVA-correction and with PVA-correction) with age (UKBB cohort).

Figures

Figure S1: Brief summary outline illustrating the three datasets used in the study.

Figure S2: Representative CBF maps for a participant from the ACP cohort.

Figure S3: Relationship between average PVA-corrected regional CBF between the ACP and UKBB cohorts (controls). Voxel-wise CBF values were predicted using voxel-wise rsfMRI timeseries data first and then signals were averaged from corresponding voxels to get the average regional CBF values.

Figure S4: Relationship between the correlation values for gray matter (GM) thickness vs. age and the correlation values for predicted regional CBF values (no PVA-corrected and PVA-corrected) vs. age.

Figure S5: Relationship between noise signals from the raw rsfMRI data using MPPCA denoising technique and the corresponding band-wise spectral power density measures (*N*=40).

Figure S6: Association between the average predicted rCBF values (DK atlas) from the UKBB healthy controls and the correspondingly measured rCBF values from the Amen Clinics Inc. healthy controls.

Materials and Methods

Study samples

ACP: ACP cohort shares a similar rural upbringing and lifestyle that includes the same level of basic school education, diet, and occupations, and virtually no illicit substance use. The cohort offers a population-level reduction in environmental heterogeneity that may confound cerebral blood flow measures. For this analysis, the primary exclusion criteria to avoid confounded effects of illnesses included major medical and neurological conditions that might affect gross brain structures - such as developmental disability, head trauma, seizure, stroke, or transient ischemic attack, and major psychiatric illnesses.

UKBB: We separated this sample into MDD cases and controls. The MDD group consisted of N=2,290 participants (790 M/1,500 F, mean age ± s.d.: 62.1±7.4 years) with recurrent MDD. Subjects in the recurrent MDD sample experienced on average 3.5 major depressive episodes in their lifetime. N=6,106 subjects were free of MDD and any other psychiatric condition (2,287 M/3,819 F, mean age ±s.d.: 61.9±7.1 years), considered as controls in this study. We took a conservative approach to defining groups so that N=10,502 participants were left unclassified because of the lack of clinical records leading to a definitive conclusion on certain criteria, such as who had metabolic, neurological and psychiatric conditions (including bipolar disorder), stroke, cardiac condition or brain cancer, per hospital records or self-report (1). The university of Maryland, Baltimore provided the initial ethical approval for using the UKBB datasets.

Arterial spin labeling data acquisition, processing, and CBF extraction

In the ACP, the arterial-spin labeling (ASL) data were acquired on a 3 T Siemens Prisma scanner with 64 channels, using three-dimensional (3D) pseudo-continuous ASL (pCASL) with background suppressed gradient and a spin-echo sequence consisting of 13 pairs of labeled and control scans. The acquisition parameters were 2.5 mm isotropic spatial resolution, matrix size=96×96 with 58 axial slices, repetition time/echo time (TR/TE) =4,000/37 ms, flip angle=120°, field of view (FoV) read=220 mm, FoV phase=100%, post-label delay=1700 ms, labeling duration=1650 ms. Total scan time was approximately 10 minutes. A 3D T_1-weighted image was acquired for anatomical reference, as well as gray and white matter tissue segmentation. A volume of M_0 image was also acquired without background suppression to normalize the control-label difference for cerebral blood flow (CBF) quantification. To suppress effects of noise, the M_0 image was smoothed with a 5×5×5 mm^3 Gaussian-kernel (2).

CBF data analysis was performed with the FSL software package; perfusion was estimated by using a standard single-compartment ASL model; partial volume effects correction was performed with a spatially regularized method (3). Spatial regularization, motion correction and partial volume corrections were performed in FSL v6.0.1. The high- resolution structural image provided partial volume estimates (PVE) for the different tissue types (gray matter (GM), white matter (WM), and cerebrospinal fluid (CSF)). The high-resolution PVE images obtained from a structural image were then converted to the ASL image space using a transformation matrix from the structural space to the ASL native image space. Partial volume corrected CBF maps were used to extract the quantitative voxelwise CBF signals using volumetric Desikan-Killiany (DK) atlas that consisted of thirty-four cortical brain regions from each hemisphere.

RsfMRI data acquisition, processing, time series extraction, and spectral features

ACP participants underwent rsfMRI data acquisition that consisted of two runs. Oblique axial acquisitions alternated between phase encoding in the anterior-to-posterior (AP) and posterior-to-anterior (PA) directions within a single run. Separate single-band reference images, acquired for phase encoding in AP and PA directions, were used for spatial distortion correction. RsfMRI data were acquired using the following parameters: TR=780 ms, TE=34.4 ms, spatial resolution of 2-mm isotropic voxels, matrix size=104×104 with 72 axial slices, number of volumes=420, flip angle=52°, multi-band acceleration factor=8, and bandwidth=2,186 Hz/pixel. Total rsfMRI scan duration was 11 min and 14 sec (5 min and 37 sec for both AP and PA direction each).

UKBB rsfMRI data were acquired on 3T Siemens Skyra scanners with the standard Siemens 32-channel receive head coil using the following parameters: TR=735 ms, TE=39 ms, spatial resolution of 2.4-mm isotropic voxels, matrix size=88×88 with 64 axial slices, number of volumes=490, flip angle=52°, and multi-band acceleration factor=8. A separate single-band reference image was acquired and used as the reference scan for head motion correction and alignment to other modalities (4). Total rsfMRI scan duration was 6 min and 10 sec.

The resting state analysis workflow developed by the Enhancing Neuro Imaging Genetics through Meta-Analysis (ENIGMA) consortium was used to process the rsfMRI data; processing steps have been described in detail in prior publications (5, 6). The analysis workflow uses Marchenko-Pastur principal components analysis denoising (7) to improve signal-to noise ratio (SNR)/temporal SNR of the time series data. In this workflow, a transformation is computed, registering the base volume to the ENIGMA EPI template, which is used as a common anatomical spatial reference frame for registration purposes. This step was followed by 3D

deconvolution of methodological covariates, and regression of the global signal. Each functional volume was registered to the volume with the minimum outlier fraction for head motion correction, where each transformation was concatenated with the transformation to standard space, to avoid unnecessary interpolation. We removed the effects of the nuisance variables (the motion parameters and their temporal derivatives, and time courses from the local WM and CSF from lateral ventricles) by including them as covariates in the multiple linear regression analysis. Motion was estimated as the magnitude of displacement from one time point to the next including neighboring time points. Time points, with excessive motion (>0.2 mm) and those points which included more than 0.1 outlier voxels fraction, were excluded from further analysis. Images were spatially normalized to the ENIGMA EPI template in 2 mm isotropic MNI standard space for group analysis.

Structural MRI data collection and partial voxel occupancy calculation

ACP T_1-weighted imaging. High-resolution (0.8 mm isotropic), high GM-WM contrast (~25%) T_1-weighted images were acquired using a retrospective motion-corrected protocol using 3T Siemens Prisma scanner equipped with a 64-channel head coil. With this protocol, four full-resolution volumes are acquired using a T_1-weighted, 3D MPRAGE sequence with an adiabatic inversion contrast pulse with the following scan parameters: TR=2,400 ms, TE=2.22 ms, inversion time=1,000 ms, flip angle=8°, matrix=300×320, slices per slab=208, and a spatial resolution =0.80 mm isotropic.

Supplementary Tables

Region	Abbreviation	MDD effect size for predicted rCBF without PVA-correction (UKBB)
Banks of superior temporal sulcus	BSTS	-0.33 ($p<10^{-16}$)
Caudal anterior cingulate	CACG	-0.36 ($p<10^{-16}$)
Caudal middle frontal gyrus	CMFG	-0.10 ($p=4\times10^{-5}$)
Cuneus	CU	-0.33 ($p<10^{-16}$)
Entorhinal cortex	EC	-0.23 ($p=3\times10^{-8}$)
Fusiform gyrus	FG	-0.36 ($p<10^{-16}$)
Inferior parietal gyrus	IPG	-0.34 ($p<10^{-16}$)
Inferior temporal gyrus	ITG	-0.31 ($p<10^{-16}$)
Isthmus cingulate gyrus	ICG	-0.35 ($p<10^{-16}$)
Lateral occipital gyrus	LOG	-0.37 ($p<10^{-16}$)
Lateral orbito-frontal gyrus	LOFG	-0.42 ($p<10^{-16}$)
Lingual gyrus	LG	-0.37 ($p=10^{-16}$)
Medial orbito-frontal gyrus	MOFG	-0.38 ($p<10^{-16}$)
Middle temporal gyrus	MTG	-0.38 ($p<10^{-16}$)
Para hippocampal gyrus	PHIG	-0.30 ($p<10^{-16}$)
Para central gyrus	PaCG	-0.30 ($<10^{-16}$)
Pars-opercularis	POP	-0.43($<10^{-16}$)
Pars-orbitalis	POR	-0.29 ($p<10^{-16}$)
Pars-triangularis	PTR	-0.28 ($p<10^{-16}$)
Pericalcarine	PCAL	-0.29 ($p<10^{-16}$)
Postcentral gyrus	PoCG	-0.31 ($p<10^{-16}$)
Posterior cingulate gyrus	PCG	-0.38 ($p<10^{-16}$)
Precentral gyrus	PrCG	-0.33 ($p<10^{-16}$)
Precuneus	PCU	-0.30 ($p<10^{-16}$)
Rostral anterior cingulate gyrus	RACG	-0.05 ($p=0.04$)
Rostral middle frontal gyrus	RMFG	-0.27 ($p<10^{-16}$)
Superior frontal gyrus	SFG	-0.30 ($p<10^{-16}$)
Superior parietal gyrus	SPG	-0.30 ($p<10^{-16}$)
Superior temporal gyrus	STG	-0.36 ($p<10^{-16}$)
Supramarginal gyrus	SMG	-0.34 ($p<10^{-16}$)
Frontal pole	FP	-0.09 ($p=2\times10^{-4}$)
Temporal pole	TP	-0.30 ($p<10^{-16}$)
Transverse temporal gyrus	TTG	-0.33 ($p<10^{-16}$)
Insula	IN	-0.35 ($p<10^{-16}$)

Table S1. Regional MDD effect size for predicted rCBF values with no PVA-correction for the UKBB cohort.

Region	*Abbreviation*	*Correlation between cortical thickness with age*	*MDD effect size for cortical thickness (UKBB)*
Banks of superior temporal sulcus	BSTS	-0.21	0.00 ($p=6\times10^{-1}$)
Caudal anterior cingulate	CACG	-0.08	0.00 ($p=5\times10^{-1}$)
Caudal middle frontal gyrus	CMFG	-0.31	-0.03 ($p=4\times10^{-2}$)
Cuneus	CU	-0.14	-0.03 ($p=4\times10^{-2}$)
Entorhinal cortex	EC	-0.16	-0.03 ($p=1\times10^{-1}$)
Fusiform gyrus	FG	-0.25	-0.04 ($p=8\times10^{-3}$)
Inferior parietal gyrus	IPG	-0.24	-0.01 ($p=4\times10^{-1}$)
Inferior temporal gyrus	ITG	-0.17	-0.01 ($p=4\times10^{-1}$)
Isthmus cingulate gyrus	ICG	-0.17	-0.01 ($p=8\times10^{-1}$)
Lateral occipital gyrus	LOG	-0.06	0.04 ($p=1\times10^{-1}$)
Lateral orbito-frontal gyrus	LOFG	-0.13	-0.02 ($p=3\times10^{-1}$)
Lingual gyrus	LG	-0.10	0.01 ($p=3\times10^{-1}$)
Medial orbito-frontal gyrus	MOFG	-0.15	-0.04 ($p=2\times10^{-2}$)
Middle temporal gyrus	MTG	-0.23	-0.05 ($p=1\times10^{-2}$)
Para hippocampal gyrus	PHIG	-0.13	-0.01 ($p=2\times10^{-1}$)
Paracentral gyrus	PaCG	-0.29	-0.02 ($p=1\times10^{-1}$)
Pars-opercularis	POP	-0.30	-0.05 ($p=2\times10^{-3}$)
Pars-orbitalis	POR	-0.24	-0.04 ($p=11\times10^{-1}$)
Pars-triangularis	PTR	-0.33	-0.03 ($p=1\times10^{-1}$)
Pericalcarine	PCAL	-0.12	0.06 ($p=3\times10^{-3}$)
Postcentral gyrus	PoCG	-0.27	0.00 ($p=7\times10^{-1}$)
Posterior cingulate gyrus	PCG	-0.17	-0.01 ($p=4\times10^{-1}$)
Precentral gyrus	PrCG	-0.22	-0.03 ($p=3\times10^{-2}$)
Precuneus	PCU	-0.31	-0.02 ($p=2\times10^{-1}$)
Rostral anterior cingulate gyrus	RACG	-0.12	-0.04 ($p=4\times10^{-2}$)
Rostral middle frontal gyrus	RMFG	-0.35	-0.04 ($p=5\times10^{-2}$)
Superior frontal gyrus	SFG	-0.39	-0.07 ($p=2\times10^{-4}$)
Superior parietal gyrus	SPG	-0.24	0.03 ($p=5\times10^{-1}$)
Superior temporal gyrus	STG	-0.25	0.00 ($p=1\times10^{-1}$)
Supramarginal gyrus	SMG	-0.14	-0.05 ($p=2\times10^{-3}$)
Frontal pole	FP	-0.18	-0.02 ($p=6\times10^{-1}$)
Temporal pole	TP	-0.12	---
Transverse temporal gyrus	TTG	0.11	0.04 ($p=3\times10^{-1}$)
Insula	IN	-0.14	-0.02 ($p=2\times10^{-1}$)

Table S2. Correlation between cortical thickness with age and MDD effect size for cortical thickness (UKBB cohort).

Region	Abbreviation	Correlation between predicted CBF (no PVA-correction) with age	Correlation between predicted CBF (PVA-correction) with age
Banks of superior temporal sulcus	BSTS	-0.04, $p=5\times10^{-4}$	-0.05, $p=4\times10^{-6}$
Caudal anterior cingulate	CACG	-0.05, $p=2\times10^{-5}$	-0.06, $p=1\times10^{-8}$
Caudal middle frontal gyrus	CMFG	-0.11, p=2E-23	-0.13, $p=6\times10^{-35}$
Cuneus	CU	-0.05, $p=8\times10^{-7}$	-0.11, $p=8\times10^{-24}$
Entorhinal cortex	EC	-0.03, $p=2\times10^{-3}$	-0.20, $p=3\times10^{-75}$
Fusiform gyrus	FG	-0.05, $p=1\times10^{-5}$	-0.25, $p=1\times10^{-120}$
Inferior parietal gyrus	IPG	-0.02, p=0.1	0.04, $p=5\times10^{-5}$
Inferior temporal gyrus	ITG	0.02, p=0.07	-0.05, $p=5\times10^{-6}$
Isthmus cingulate gyrus	ICG	-0.09, $p=1\times10^{-16}$	-0.10, $p=2\times10^{-20}$
Lateral occipital gyrus	LOG	-0.04, $p=6\times10^{-5}$	-0.05, $p=2\times10^{-5}$
Lateral orbito-frontal gyrus	LOFG	0.02, p=0.03	-0.08, $p=3\times10^{-13}$
Lingual gyrus	LG	-0.06, $p=6\times10^{-9}$	-0.15, $p=2\times10^{-43}$
Medial orbito-frontal gyrus	MOFG	-0.01, p=0.2	-0.07, $p=4\times10^{-10}$
Middle temporal gyrus	MTG	0.02, p=0.2	0.04, $p=4\times10^{-4}$
Para hippocampal gyrus	PHIG	0.03, p=0.01	-0.07, $p=2\times10^{-11}$
Para central gyrus	PaCG	-0.03, p=0.02	-0.08, $p=3\times10^{-13}$
Pars-opercularis	POP	-0.12, $p=2\times10^{-26}$	-0.13, $p=3\times10^{-35}$
Pars-orbitalis	POR	-0.06, $p=5\times10^{-9}$	-0.08, $p=7\times10^{-14}$
Pars-triangularis	PTR	-0.07, $p=1\times10^{-11}$	-0.08, $p=8\times10^{-15}$
Pericalcarine	PCAL	-0.07, $p=1\times10^{-9}$	-0.12, $p=2\times10^{-30}$
Postcentral gyrus	PoCG	0.00, p=0.7	0.01, p=0.6
Posterior cingulate gyrus	PCG	-0.07, $p=3\times10^{-11}$	-0.11, $p=1\times10^{-25}$
Precentral gyrus	PrCG	-0.03, p=0.01	-0.08, $p=3\times10^{-14}$
Precuneus	PCU	-0.02, p=0.2	-0.04, $p=1\times10^{-4}$
Rostral anterior cingulate gyrus	RACG	-0.07, $p=2\times10^{-10}$	-0.03, p=0.008
Rostral middle frontal gyrus	RMFG	-0.11, $p=5\times10^{-24}$	-0.08, $p=2\times10^{-14}$
Superior frontal gyrus	SFG	-0.13, $p=5\times10^{-35}$	-0.17, $p=3\times10^{-55}$
Superior parietal gyrus	SPG	-0.01, p=0.3	0.02, p=0.07
Superior temporal gyrus	STG	0.00, p=0.8	0.05, $p=1\times10^{-6}$
Supramarginal gyrus	SMG	-0.01, p=0.5	0.01, p=0.60
Frontal pole	FP	-0.05, $p=2\times10^{-5}$	-0.04, $p=4\times10^{-4}$
Temporal pole	TP	-0.05, $p=4\times10^{-6}$	-0.17, $p=2\times10^{-55}$
Transverse temporal gyrus	TTG	0.10, $p=4\times10^{-20}$	0.20, $p=1\times10^{-76}$
Insula	IN	0.03, p=0.02	-0.08, $p=6\times10^{-12}$

Table S3. Correlation coefficients between predicted CBF (no PVA-correction and with PVA-correction) with age (UKBB cohort).

Supplementary Figures

The figure S1 outlines the three datasets used in the study along with the available data and what they are used for and number of participants included in the study. Data were processed with the standardized analysis pipelines that differed based on the data (e.g. resting-state fMRI, ASL, SPECT). After preprocessing, the measures were extracted from the cortical brain regions using Desikan-Killiany atlas for further comparison between these measures.

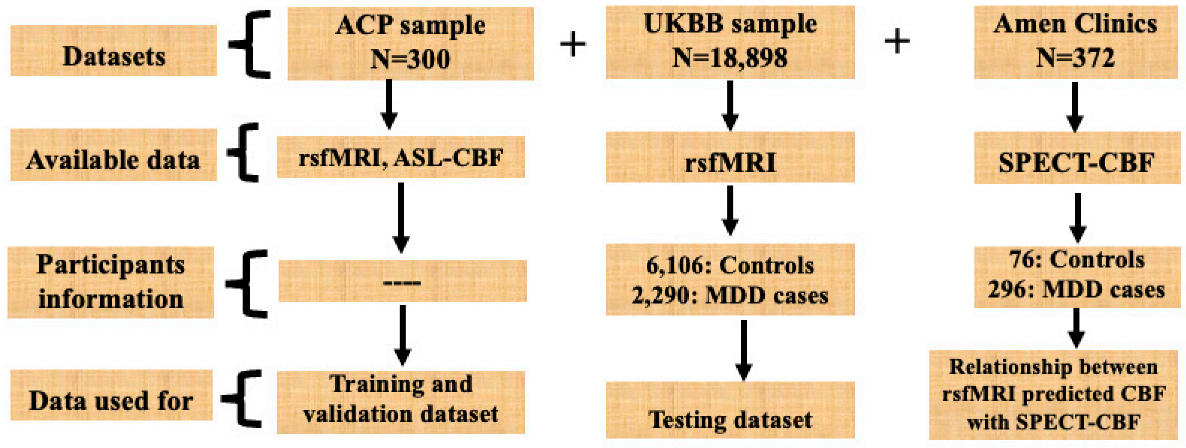

Figure S1: Brief summary outline illustrating the three datasets used in the study.

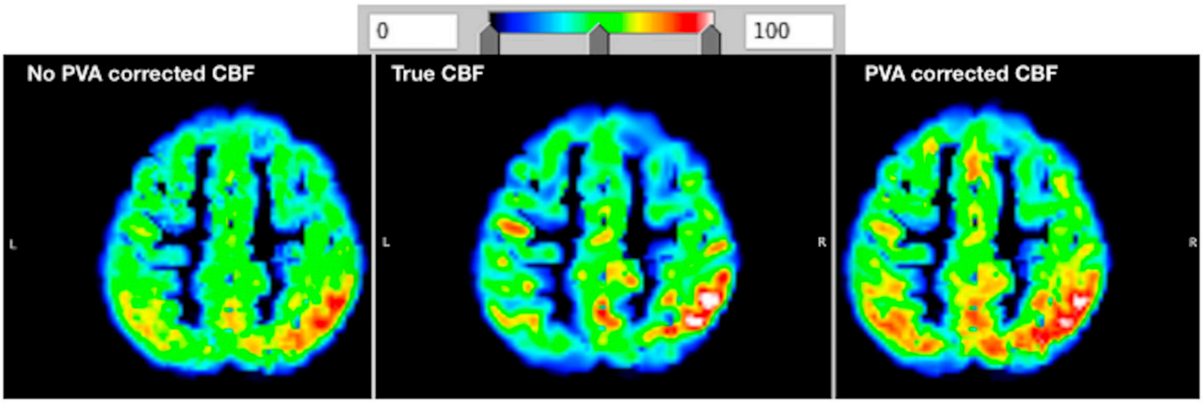

Figure S2. Representative CBF maps for a participant from the ACP cohort, CBF is measured in mL/100 g/min.

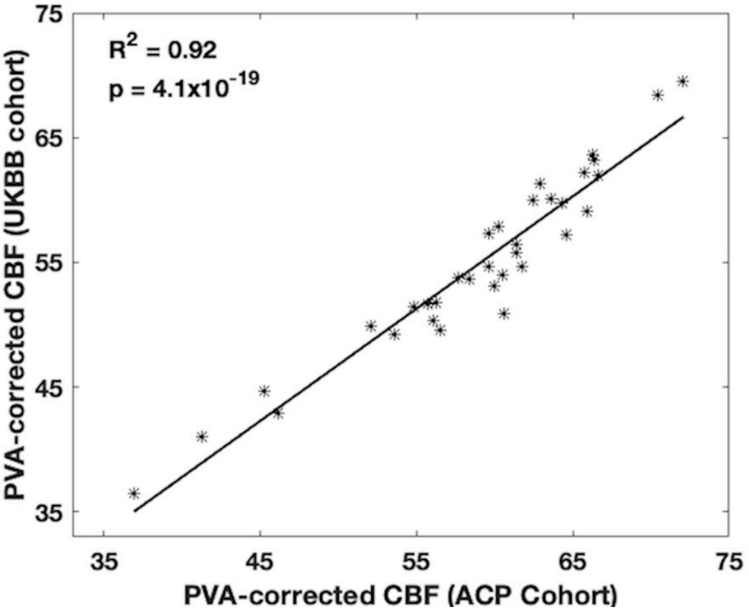

Figure S3: Relationship between average PVA-corrected regional CBF between ACP and UKBB cohorts (controls). Voxel-wise CBF values were predicted using voxel-wise rsfMRI timeseries data first and then signals were averaged from corresponding voxels to get the average regional CBF values.

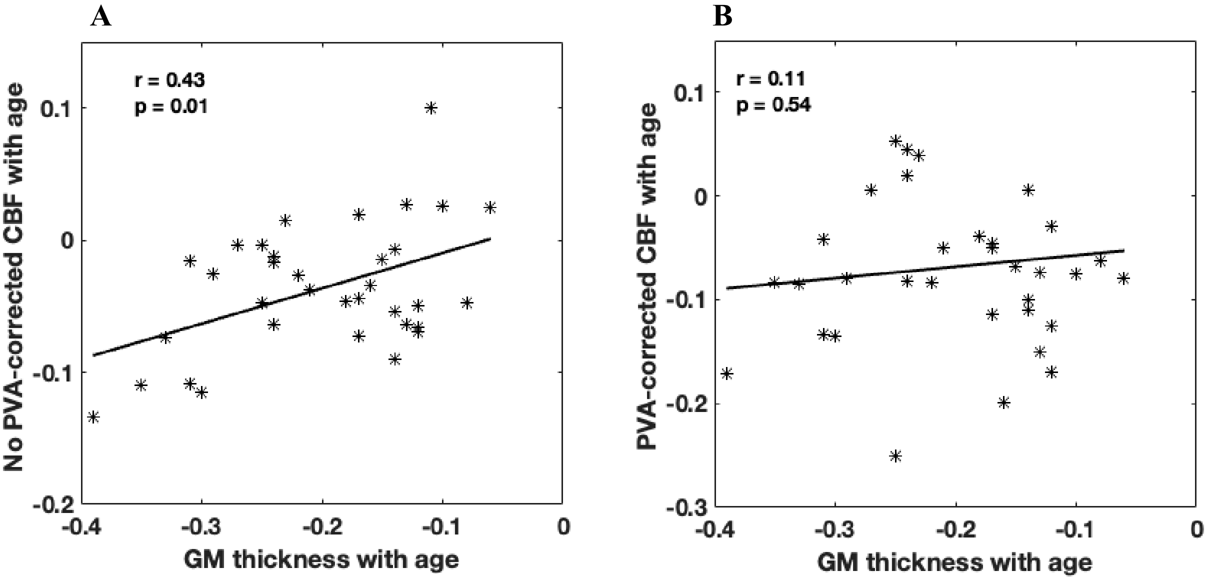

Figure S4: Relationship between the correlation values for gray matter (GM) thickness vs. age and the correlation values for predicted regional CBF values (no PVA-corrected and PVA-corrected) vs. age.

Computed the noise signals from the raw rsfMRI data (*N*=40) using MPPCA denoising technique and the corresponding band-wise spectral power density measures showed neither of the band-wise spectral power density measures had the statistically significant correlation with noise signals (**Figure S5**). This showed that the respiratory and cardiac cycles had no effects or very negligible effects in our research findings. The effects of physiological noise components could have been corrected if physiological recordings for respiratory and cardiac cycles were available.

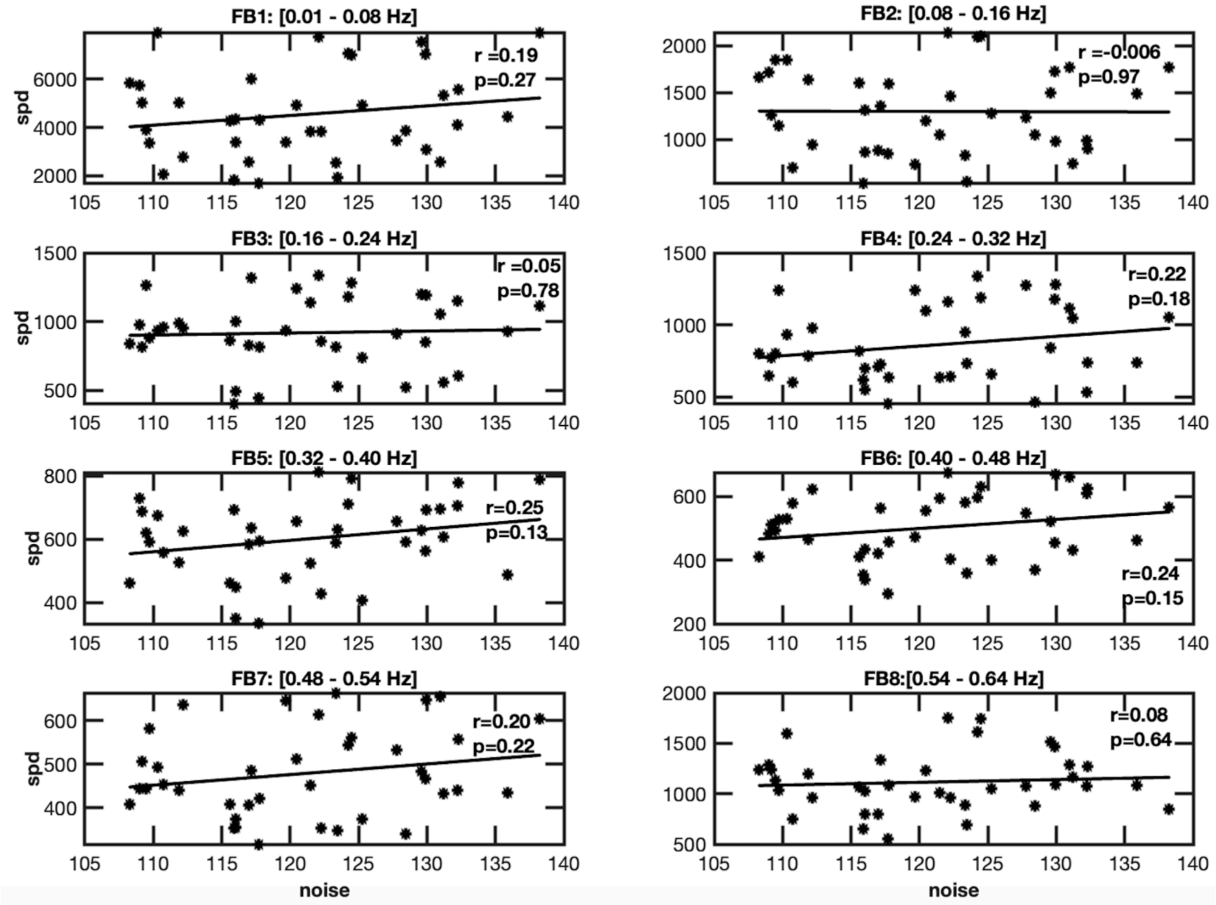

Figure S5: Relationship between noise signals from the raw rsfMRI data using MPPCA denoising technique and the corresponding band-wise spectral power density measures (*N*=40).

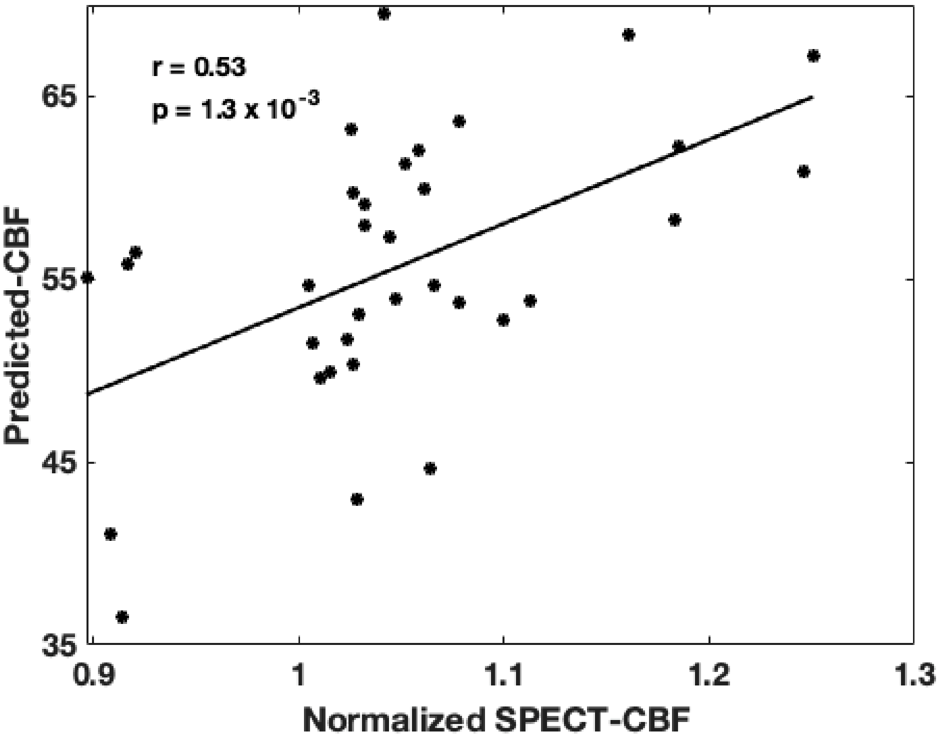

Figure S6.

The average predicted rCBF values (extracted using the DK cortical brain regions) from the UKBB healthy controls and the corresponding rCBF measures from the Amen Clinics healthy controls were found to have a significant positive correlation (r=0.53, p=1.3×10^{-3}) as in

Figure S6: Association between the average predicted rCBF values (DK atlas) from the UKBB healthy controls and the correspondingly measured rCBF values from the Amen Clinics Inc. healthy controls.

1. D. J. Smith *et al.*, Prevalence and characteristics of probable major depression and bipolar disorder within UK biobank: cross-sectional study of 172,751 participants. *PLoS One* **8**, e75362 (2013).
2. D. C. Alsop *et al.*, Recommended Implementation of Arterial Spin Labeled Perfusion MRI for Clinical Applications: A consensus of the ISMRM Perfusion Study Group and the European Consortium for ASL in Dementia. *Magn Reson Med* **73**, 102-116 (2015).
3. M. A. Chappell *et al.*, Partial volume correction of multiple inversion time arterial spin labeling MRI data. *Magn Reson Med* **65**, 1173-1183 (2011).
4. F. Alfaro-Almagro *et al.*, Image processing and Quality Control for the first 10,000 brain imaging datasets from UK Biobank. *NeuroImage* **166**, 400-424 (2018).
5. B. M. Adhikari *et al.*, Heritability estimates on resting state fMRI data using ENIGMA analysis pipeline. *Pac Symp Biocomput* **23**, 307-318 (2018).
6. B. M. Adhikari *et al.*, Comparison of heritability estimates on resting state fMRI connectivity phenotypes using the ENIGMA analysis pipeline. *Hum Brain Mapp.* **39**, 4893-4902 (2018).
7. J. Veraart, E. Fieremans, D. S. Novikov, Diffusion MRI noise mapping using random matrix theory. *Magn Reson Med.* **76**, 1582-1593 (2016).
